# 4-Hydroxyhexenal- and 4-Hydroxynonenal-Modified Proteins in Pterygia

**DOI:** 10.1155/2013/602029

**Published:** 2013-05-12

**Authors:** Ichiya Sano, Sachiko Kaidzu, Masaki Tanito, Katsunori Hara, Tsutomu Okuno, Akihiro Ohira

**Affiliations:** ^1^Department of Ophthalmology, Shimane University Faculty of Medicine, Enya 89-1, Izumo, Shimane 693-8501, Japan; ^2^National Institute of Occupational Safety and Health, Kawasaki, Japan

## Abstract

Oxidative stress has been suspected of contributing to the pathogenesis of pterygia. We evaluated the immunohistochemical localization of the markers of oxidative stress, that is, the proteins modified by 4-hydroxyhexenal (4-HHE) and 4-hydroxynonenal (4-HNE), which are reactive aldehydes derived from nonenzymatic oxidation of n-3 and n-6 polyunsaturated fatty acids, respectively. In the pterygial head, labeling of 4-HHE- and 4-HNE-modified proteins was prominent in the nuclei and cytosol of the epithelium. In the pterygial body, strong labeling was observed in the nuclei and cytosol of the epithelium and proliferating subepithelial connective tissue. In normal conjunctival specimens, only trace immunoreactivity of both proteins was observed in the epithelial and stromal layers. Exposures of ultraviolet (330 nm, 48.32 ± 0.55 J/cm^2^) or blue light (400 nm, 293.0 ± 2.0 J/cm^2^) to rat eyes enhanced labeling of 4-HHE- and 4-HNE-modified proteins in the nuclei of conjunctival epithelium. Protein modifications by biologically active aldehydes are a molecular event involved in the development of pterygia.

## 1. Introduction

Pterygium, a common ocular surface disease only observed in humans [1, 2], is a chronic condition characterized by encroachment of a fleshy, triangular portion of the bulbar conjunctiva into the cornea. Pterygia develop more often on the nasal side of the eye and are often bilateral [2–6]. Progression of lesions with central migration into the visual axis results in severe visual impairment [7, 8]; thus, surgical excision needs to be considered when progression occurs. 

Histologically, pterygia are comprised of a superficial growth of a highly vascularized elastoid and basophilic degenerated connective tissue; that is, covered by an alternately thickened or thinned epithelium [9]. Although the pathogenesis of pterygia is not fully understood, it has been proposed that their typical location can be explained by the corneal focusing of incident sunlight on the medial limbus [10–12]. Epidemiologic studies also have indicated a possible association between chronic sunlight exposure, especially ultraviolet light (UV), and development of pterygia [2, 13–15]. The detrimental effects of ultraviolet irradiation could be due either directly to an ultraviolet phototoxic effect or indirectly to formation of radial oxygen species that cause oxidative stress [16].

The current thinking suggests that free radicals that form during oxidative stress can directly attack critical biomolecules including polyunsaturated fatty acids (PUFAs) and initiate free radical chain reactions that result in lipid peroxidation in cellular membranes. This chain reaction amplifies generation of lipid radical species, causing PUFA degeneration in a variety of oxidized products, including aldehydes [17]. 4-Hydroxyhexenal (4-HHE) and 4-hydroxynonenal (4-HNE) are *α*, *β*-unsaturated aldehydes that are end products of nonenzymatic oxidation of n-3 and n-6 polyunsaturated fatty acids, respectively [18]. These highly reactive aldehydes can react readily with histidine, cysteine, or lysine residues of proteins, leading to formation of stable Michael adducts with a hemiacetal structure [19]. Formation of these adducts leads to a variety of cytopathological effects, that is, inhibition of enzyme activity; inhibition of protein, RNA, and DNA synthesis; cell cycle arrest, and apoptosis [20, 21]. The use of specific antibodies to recognize the hemiacetal structure of the Michael adducts enables their detection in tissues [22].

Increasing evidence suggests that protein modifications by reactive aldehydes are involved in various diseases. We evaluated the immunohistochemical localization of proteins modified by these aldehydes in pterygial specimens from patients and conjunctivae from rats that were exposed to the short wavelength lights. The aims of the current study were to evaluate the expression of the two aldehyde-modified proteins in the epithelial and stromal layers of human pterygia and normal conjunctiva to determine if they participate in the development of pterygia, and to test the possible relationship between short-wavelength light radiation and the development of pterygia. 

## 2. Subjects and Methods

Human biopsy study was conducted as a part of the study protocol “Establishment of a Library of Ocular Tissues and Cells Obtained during Various Ophthalmic Surgeries,” that the institutional review board of Shimane University Hospital reviewed and approved. All subjects provided written informed consent. The human biopsy specimens of pterygia and adjacent normal conjunctiva were obtained intraoperatively during pterygial excision from four eyes of three patients (1 man, 2 women; age range, 63–80 years). 

The specimens were fixed in 4% paraformaldehyde containing 20% isopropanol, 2% trichloroacetic acid, and 2% zinc chloride, for 24 hours at room temperature, processed for paraffin embedding, and morphologically analyzed using hematoxylin and eosin (H&E) staining and immunohistochemistry for 4-HHE and 4-HNE, as described previously [23, 24]. Briefly, the sections were deparaffinized and endogenous peroxidase activity was inactivated with 3% H_2_O_2_ for 10 minutes. After blocking with a serum-free blocking reagent (Dako, Carpinteria, CA, USA) for 30 minutes at room temperature, the sections were incubated with the anti-4-HHE (1 : 100) or anti-4-HNE (1 : 100) antibody diluted with antibody diluent (Dako) for 2 hours at 37°C and then with the peroxidase-linked anti-mouse IgG polymer (EnVision+ System, Dako) for 1 hour at 37°C. For negative control experiments, sections were incubated with antibody diluent without a primary antibody for 2 hours at 37°C and then with the peroxidase-linked anti-mouse IgG polymer for 1 hour at 37°C. Signals were developed with 3′,3′-diaminobenzidine (Dako) in chromogen solution. Monoclonal anti-4-HHE- and anti-4-HNE-modified protein antibodies were purchased from NOF Corporation (Tokyo, Japan).

For animals, all procedures were performed according to the ARVO Statement for the Use of Animals in Ophthalmic and Vision Research and The Shimane University Guidelines for Animals in Research. Male Sprague-Dawley rats (4-week-old) were obtained from Charles River Laboratories Japan Inc. (Kanagawa, Japan) and maintained in our colony room for 7–10 days before the experiments. The light intensity in the cages was 10–20 lux. All rats were kept in a 12-hour (7 AM to 7 PM) light-dark cycle.

After anesthesia was induced by the intramuscular injection of a mixture of ketamine (120 mg/kg) and xylazine (6 mg/kg), light exposure was performed to the left eyes, and the opposite eyes left unexposed to light were served as controls. Rats were exposed to 330 or 400 nm lights with 10 nm in bandwidth, using a xenon lamp light source with bandpass filters (Asahi Spectra Co., Ltd., Tokyo, Japan) for an estimated period as described later. Light was exposed at a right angle to the center of the cornea. During exposure, diluted saline (×2) was adequately dropped to the surface of the cornea to prevent drying, and intramuscular injection of anesthetic drug was added to keep the anesthesia. After exposure, rats were kept in the cyclic light (10–20 lux, 12-hour light-dark cycle) for 7 days until enucleation. The number of animals was 6 (12 eyes) in each light wavelength.

Before the start of each exposure, irradiance was measured at the position of the cornea with a radiometer (IL 1400A, International Light Technologies, Peabody, MA, USA) connected to a silicon photodiode detector (SEL033, International Light Technologies), and exposure duration was determined by dividing the target corneal radiant exposure. The radiometer was calibrated prior to each light exposure. After the light exposure, corneal irradiance measured again, and with the initial measurement value was recalculated to secure the radiant exposure to the cornea was around 48.32 ± 0.55 J/cm^2^ for 330 nm and 293.0 ± 2.0 J/cm^2^ for 400 nm light.

The eyes enucleated were analyzed by H&E and immunohistochemical procedures as described in human specimen analysis.

## 3. Results and Discussion

**Figure 1:**
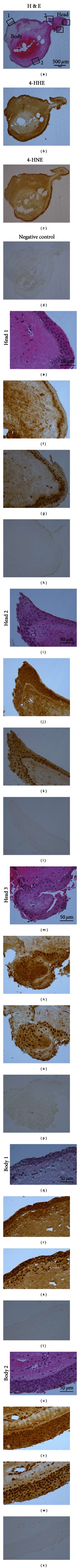
Expressions of aldehyde-modified proteins in pterygia. Representative images of H&E staining (a, e, i, m, q, and u), 4-HHE (b, f, j, n, r, and v) and 4-HNE (c, g, k, o, s, and w) immunohistochemistry, and negative control staining (d, h, l, p, t, and x) are shown. Three specimens from the pterygial head (head 1, 2, and 3) and two specimens from the pterygial body (body 1 and 2) are shown at higher magnifications. H&E: hematoxylin and eosin; 4-HHE: 4-hydroxyhexenal; 4-HNE: 4-hydroxynonenal.

**Figure 2:**
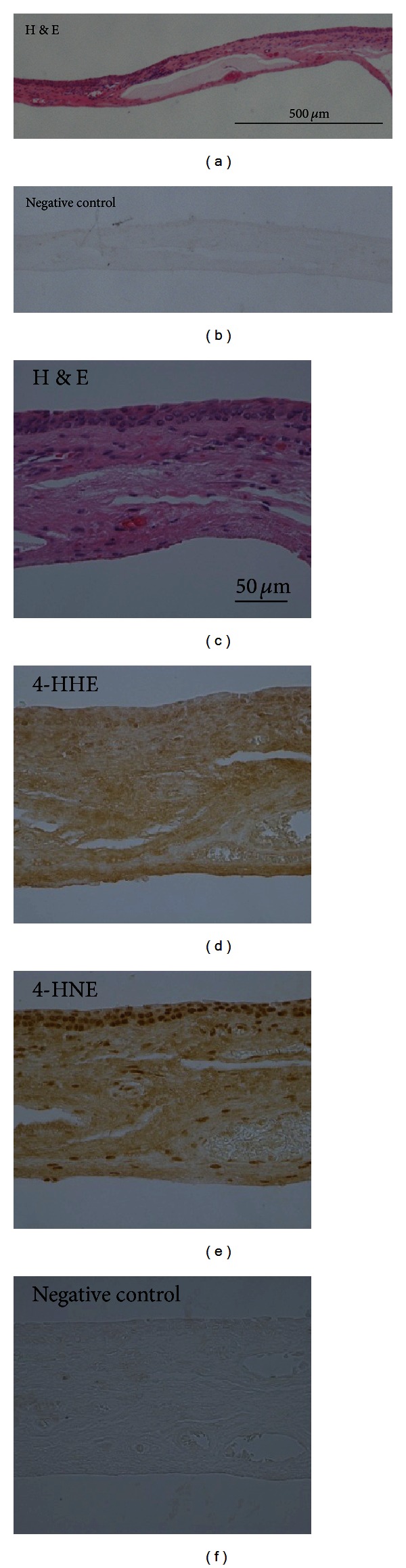
Expressions of aldehyde-modified proteins in normal conjunctiva. Representative images of H&E (a, c) staining, 4-HHE (d) and 4-HNE (e) immunohistochemistry, and negative control staining (b, f) are shown. (a, b) Lower magnification. (c–f) Higher magnification. H&E: hematoxylin and eosin; 4-HHE: 4-hydroxyhexenal; 4-HNE: 4-hydroxynonenal.

At lower magnifications (Figures 1(a)–1(d)), the 4-HHE- and 4-HNE-modified proteins were seen throughout the pterygial specimens including the head, where the corneal-migration front of the pterygium, and the body, where active proliferation of subepithelial connective tissues occurred. At higher magnifications, in the head of the pterygium (Figures 1(e)–1(p)), prominent immunoreactivity of both 4-HHE- and 4-HNE-modificed proteins was observed in the nuclei and cytosol of the epithelium; the immunoreactivity of both aldehyde-modified proteins was moderate in the subepithelial stroma. In the body (Figures 1(q)–1(x)), strong immunoreactivity of the aldehyde-modified proteins was observed in the nuclei and cytosol of the epithelium and in the subepithelial stromal layer. The expression patterns of both aldehyde-modified proteins were consistent in three other pterygial specimens analyzed (data not shown). In the normal conjunctival specimen, only trace immunoreactivity was observed in the epithelial and stromal layers (Figures 2(a)–2(f)). 

The results clearly showed marked upregulation of reactive aldehydes-modified proteins in pterygial tissue compared with trace expression in normal conjunctiva; in the pterygial stromal layer, immunoreactivity was more prominent in the proliferating body than in the head. Since 4-HNE modulates cellular proliferation and differentiation including proto-oncogene expression [25], reactive aldehyde might alter the proliferative regulation in the pathogenesis of pterygium. Previously, reduced enzymatic activity of antioxidant enzymes including catalase, superoxide dismutase (SOD), and glutathione peroxidase (GPX) was reported in human pterygial tissue [16]. Protein modification by incubation with reactive aldehydes including 4-HHE and 4-HNE effectively diminishes the enzymatic activities of SOD, GPX, and glutathione S-transferase [26]. Protein modification of thioredoxin, another antioxidant enzyme, by 4-HNE initiates tissue inflammation [27]. Thus, a compromised antioxidative defense system and initiation of inflammation due to oxidative modification of antioxidant enzymes may be involved in the proliferative mechanisms of pterygia. 

**Figure 3:**
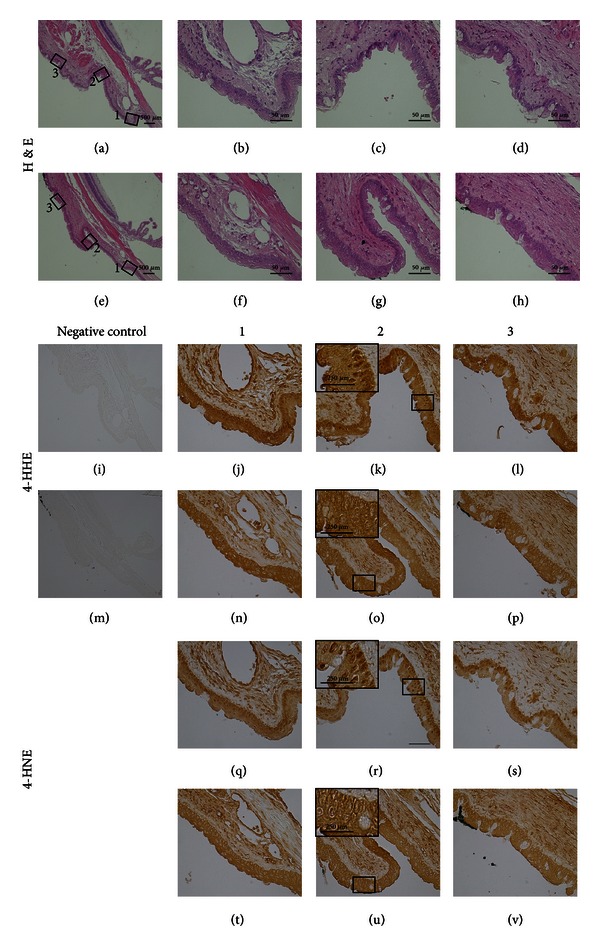
Expressions of aldehyde-modified proteins in conjunctiva from rats exposed to 330 nm wavelength light. The eyes were exposed (a–d, i–l, and q–s) or unexposed (e–h, m–p, and t–v) to the light. Representative images of H&E staining (a–h) 4-HHE (j–l, n–p) and 4-HNE (q–v) immunohistochemistry, and negative control staining (i, m) are shown. Three specimens from the conjunctiva (1, 2, and 3) are shown at high magnifications. Insertions are higher magnifications of squares (k, o, r, and u). H&E: hematoxylin and eosin; 4-HHE: 4-hydroxyhexenal; 4-HNE: 4-hydroxynonenal.

**Figure 4:**
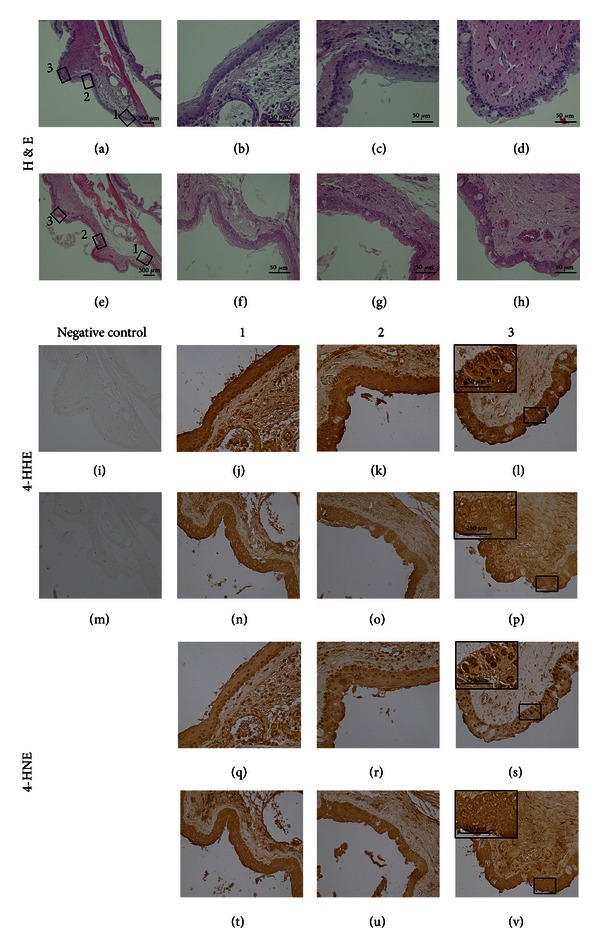
Expressions of aldehyde-modified proteins in conjunctiva from rat exposed to 400 nm wavelength light. The eyes were exposed (a–d, i–l, and q–s) or unexposed (e–h, m–p, and t–v) to the light. Representative images of H&E staining (a–h), 4-HHE (j–l, n–p) and 4-HNE (q–v) immunohistochemistry, and negative control staining (i, m) are shown. Three specimens from the conjunctiva (1, 2, and 3) are shown at high magnifications. Insertions are higher magnifications of squares (l, p, s, and v). H&E: hematoxylin and eosin; 4-HHE: 4-hydroxyhexenal; 4-HNE: 4-hydroxynonenal.

We also tested a possible association between light exposure and levels of protein modifications by 4-HNE and 4-HHE in rats' conjunctival samples. Compared to eyes unexposed to the light (Figures 3(n)–3(p), 3(t)–3(v), 4(n)–4(p), and 4(t)–4(v)), exposures of UV (Figure 3) or blue light (Figure 4) clearly enhanced the nuclear labeling of both 4-HHE-(Figures 3(j)–3(l) and 4(j)–4(l)) and 4-HNE-(Figures 3(q)–3(s) and 4(q)–4(s)) modified proteins in conjunctivae. The expression patterns of both aldehyde-modified proteins were consistent in 5 other animals analyzed (data not shown). 

Although other factors may contribute to pterygial development, intracellular damage accruing from short wavelength light including UV exposure has been suggested to be the most important likely etiology [15, 28, 29]. UV radiation acts directly by phototoxicity or indirectly through free radicals. Intracellularly, the free radicals cause oxidative damage by acting on macromolecules such as proteins, lipids, and nucleic acids [30]. Previous investigators have demonstrated the formation of 8-hydroxydeoxyguanosine, an established marker of oxidized damage in nucleic acids [31, 32] in pterygia [33, 34]. The current study found evidence that oxidation of lipids and proteins also is involved in the pathogenesis of pterygial formation and progression; the results suggest the possible causative relationships between UV or short-wavelength visible light radiation and the oxidation of lipids and proteins in pterygia. 

## 4. Conclusions 

 Oxidative stress has been suspected of contributing to the pathogenesis of pterygia. We assessed the possible relationship between abnormal protein oxidation and modification by reactive aldehydes in pterygia. The results suggested that protein modifications by 4-HNE and 4-HHE are molecular events involved in the development of pterygia and that short-wavelength light radiations to ocular surface are involved in these aldehyde-modified protein formations.
